# An exploration and description of experiences of at-risk youth in a correctional center in Eswatini regarding a resilience intervention program

**DOI:** 10.3389/fpsyg.2022.981428

**Published:** 2022-10-12

**Authors:** Sifiso B. Shabangu, Vicki Koen

**Affiliations:** Faculty of Health Sciences, School of Psychosocial Health, North-West University, Mafikeng, South Africa

**Keywords:** at-risk youth, Eswatini, intervention program, juvenile corrections, resilience

## Abstract

Resilience-focused programs advocate nurturing positive adoptive traits to inform prevention and intervention efforts. Considering that no resilience intervention programs have been developed specifically for at-risk youth in correctional centers in Eswatini, the authors developed a program with this focus through a combination of literature review and earlier empirical research with correctional officers and youth in a correctional center in Eswatini. This study aimed to evaluate the program through exploration and description of experiences of at-risk youth in a correctional center in Eswatini regarding the program. A purposive sample of 16 youths in a correctional center in Eswatini participated in a World Café, and data were analyzed thematically. Three themes were identified: Positive outcomes of participation, challenges encountered in the program, and recommendations for the program.

## Introduction

This article forms part of a (excluded for review) PhD degree study focused on developing a resilience intervention program for at-risk youth in correctional centers in Eswatini. Correctional centers housing youth are intended as developmentally appropriate spaces for rehabilitation of those deemed to have deviated from the law (Patterson, 2018). However, these are generally notorious for poor rehabilitation outcomes (Bhabha and Candea, 2016; Tanzania and Mbelwa, 2017), which is partly reflective of the rehabilitation methods employed. The shortage of resources in countries such as Eswatini compromises the objectivity of reformation and rehabilitation of offenders (Malindisa and Winterdyk, 2015; Atilola et al., 2018)—further impairing youth development (Barnert et al., 2016). In Sub-Saharan Africa (SSA), correctional facilities are notorious for being under-resourced, overcrowded and embattled with disease (Telisinghe et al., 2016). This has negative consequences for youth and the ecologies they interact with, such as mental disorders, hopelessness, and criminal engagement (Olofinbiyi et al., 2015; Farrington et al., 2016). There is a need to strengthen or foster processes and resources that will buffer or protect against these inherent risks, and resilience has been encouraged as one such process (Bali and Sharma, 2018).

### The social ecology of resilience

Historically, resilience has been presented as an individual’s ability to deal with significantly adverse factors and sustain positive development in the process (Ebersohn, 2015; Muntean and Cojocaru, 2016). The progress in research has seen a modification from an individual-ability perspective toward the significance of interactions with social contexts or resources such as family, church or school library (Theron et al., 2014; Mann and Yadav, 2016). The social ecology understanding of resilience proposes that individuals and their environments are in a constant state of interaction, specifically when it comes to availability and access to resources that contribute to buffering or protecting against significant adverse conditions (Ungar et al., 2014). The socio-ecological understanding of resilience served as the theoretical grounding in developing the resilience intervention program that the current article evaluates through exploration and description of experiences of at-risk youth in a correctional center in Eswatini who participated in the program.

### Resilience intervention programs

Intervention programs have been reported to increase the likelihood of positive outcomes (Soydan, 2010). Resilience intervention programs, in particular, have been developed in different contexts in the general population and have generally been associated with positive health outcomes (Calitz, 2018). For example, resilience intervention programs implemented within academic contexts in the USA, Iran, and South Africa were found to be beneficial for emotional resilience among children (Stallard et al., 2005); emotional intelligence of adolescents (Adibsereshki et al., 2019); and constructive adjustment to challenges for teachers (Joubert and Hay, 2019), respectively. Furthermore, individuals with a history of trauma and adverse childhood experiences post resilience intervention program participation, demonstrated new strengths and created and used interpersonal relationships as resources to buffer consequences of trauma (Davis and Paster, 2000; Chandler et al., 2015).

In South Africa, a context-developed resilience program, Khazimula, which accounted for cultural significance among participants, was implemented on non-incarcerated youth. Post-Khazimula, these youths were able to identify and access supportive resources (Theron et al., 2014). Also in South Africa, following the socio-educational afterschool intervention program aimed at building and strengthening resilience in the microsystem at the family and school level, Mampane (2017) found that this intervention served to strengthen resilience across these microsystems and decolonized social programs, which is significant in the South African context given the country’s history of significant adversity. Populations in correctional settings are vulnerable to significant adversity (Bella et al., 2010) and resilience intervention programs in this context have had positive outcomes.

For example, in an American correctional center, male youth who participated in a resilience program demonstrated improved engagement with factors associated with positive adaptation, such as identification and viewing of adults and authority figures as positive support resources (these were mainly correctional officers as family support was very poor) (Feinstein et al., 2008). In Ohio, USA, following the Resilience Adult Corrections (RAC) program, the youthful offenders demonstrated recognition of their strengths and acceptance of mistakes, enabling acceptance of responsibility for actions that landed them in corrections (Vasquez, 2000). Additionally, Feinstein et al. (2008) also carried out a resilience intervention program with juveniles based on the significance of ecological systems as was proposed by Bronfenbrenner. They concluded that as a result of the intervention, these youth demonstrated motivation for the future with career plans and also identified external supportive resources, which included correctional officers as emotional support (Feinstein et al., 2008). The significance of environmental factors regarding resilience has been the subject of several authors (DuMont et al., 2007; Markson et al., 2015) even within the African context (Skovdal and Daniel, 2012).

In a South African juvenile offenders center, a trauma group intervention, which provided trauma education, concluded that there was a need for the provision of comprehensive trauma counseling for juvenile offenders, mainly due to the correlation between recidivism and unmitigated traumatic experiences (Langa, 2007). Another study that explored the difficulties and potential benefits of a recreation intervention with adolescent offenders highlighted that one of the challenges is minimal participation of caregivers or parents (Steyn and Louw, 2012). The protective role of accessible and available interpersonal relationships to youth resilience has been affirmed by others (Eisenberg and Resnick, 2006; Poon et al., 2012). Resilience intervention programs focusing on youth in the correctional context of Eswatini have seemingly not been covered in the available literature. Furthermore, notwithstanding existing resilience intervention programs, the development of the current program was grounded in the socio-ecological theory of resilience which highlights the role and significance of contexts and resources therein. Therefore, resilience programs developed in other contexts would not account for the available and accessible resources—such as culture- and context-specific aspects unique to at-risk youth in correctional centers in Eswatini.

### The current resilience intervention program

Considering the need for resilience intervention programs specifically for at-risk youth in correctional centers in Eswatini, the authors developed a program for this population through a multi-phase, multi-method approach in earlier research that consisted of five phases outlined in Table 1.

**TABLE 1:** Phases implemented in development of the program.

Phase	Objective	Population and sampling	Data collection	Data analyses
One: Literature review.	Conducting a literature review to gain an understanding of existing resilience intervention programs.	N/A	N/A	N/A
Two: An exploration and description of correctional officers’ perceptions of resilience of at-risk youth.	Exploring and describing the perceptions of correctional officers, including general duty officers, psychological officers, social welfare officers, chaplains, nurses, educational officers and teachers, working at correctional services, regarding the resilience of at-risk youth in a correctional center in Eswatini.	Population: Correctional officers at the research site Sampling: Purposive	Demographic information form World Café	Thematic analysis
Three: An exploration and description of at-risk youths’ resilience within the context of a correctional center in Eswatini.	Exploring and describing the resilience of at-risk youth in a correctional center in Eswatini.	Population: Youth at the research site Sampling: Purposive	Demographic information form Narratives World Café	Thematic analysis
Four: The development of a preliminary resilience intervention program for at-risk youth in this context. Five: An exploration and description of the experiences of at-risk youth in a correctional center in Eswatini with regard to the preliminary resilience intervention program	Developing and piloting the preliminary resilience intervention program with youth in a correctional center in Eswatini. Exploring and describing the experiences of at-risk youth in a correctional center in Eswatini with regard to the preliminary resilience intervention program.	N/A Population: Youth at research site who participated in a preliminary resilience intervention program Sampling: Purposive	N/A World Café	N/A Thematic analysis

The first phase consisted of a literature review to understand existing resilience intervention programs and a critical review of strengths and weaknesses of available knowledge (Thorne, 2009). The literature revealed universal pathways to resilience, risk and protective factors for youth in correctional facilities, the general population and, specifically, youth in Eswatini. Program objectives included improving, building and strengthening resilience in the individual and their ecologies.

The second phase focused on exploring and describing correctional officers’ (*n* = 15) perceptions of the resilience of at-risk youth in a correctional center in Eswatini, which were explored using the World Café (Brown and Isaacs, 2001). Findings suggested that the correctional officers perceive an interplay of resilience factors at the individual, family, corrections and community levels as necessary for holistic rehabilitative care of the juveniles. The perceptions included four themes: Manifestation of resilience, protective factors to resilience, risk factors for resilience, and proposed support for youth resilience.

The third phase comprised an exploration and description of at-risk youths’ resilience within the context of a correctional center in Eswatini through individual written narratives (*n* = 41) (Riessman, 2001) and a World Café session (*n* = 25) (Brown and Isaacs, 2001). The findings revealed four main themes: Understanding of resilience, protective factors to resilience, risk factors to resilience, and youth’s recommendations for resilience.

Phase four, the development of a resilience intervention program for at-risk youth offenders in Eswatini, was informed by the findings of the first three phases. Data collected from correctional officers and youth were analyzed separately and then integrated to identify similarities or differences in the findings. The integration focused on what at-risk youth experienced as contributing to, supporting and promoting their resilience and what correctional officers perceived as doing the same. The knowledge empirically acquired from these phases contributed to the structure, scope, themes, sequence of strategies, messages, and communication channels of the program. Once developed, the preliminary program was submitted for review to a panel of experts who reviewed and recommended adjustments to the program before presenting it to the sample applicable to this article.

The fifth phase focused on an exploration and description of the experiences of at-risk youth in a correctional center in Eswatini with regard to the preliminary resilience intervention program. These experiences were explored using the World Café (Brown and Isaacs, 2001).

### Description of the program

The theme of superheroes was chosen for the program as it was considered relatable to participants. *Emachawe* is the SiSwati word for “heroes,” and the program was named: Emachawe: A resilience intervention program for youth in correctional centers in Eswatini. Table 2 presents an outline of this program as it was presented to the participants.

**TABLE 2:** Overview of preliminary resilience intervention program structure.

Session	Theme	Mission outcomes	Content focus
1	Superhero initiation (introduction)	Establish rules of engagement Get to know fellow superheroes/participants Understanding of resilience	Rules for superheroes such as respect, freedom to speak and ask, we are here to learn. Get to know superheroes *via* icebreaker games which are safe and allow for introductions. Introduce participants to socio-ecological understanding of resilience. Explain a context, significant adversity, resources, and positive adjustment.
2	The Villains: Exploration of risk factors	Identify risk factors generally and in our lives Identify contexts where risk factors exist	Identifying villains/risk factors. Explore what can get in the way of positive events or experiences. These can include poor correctional conditions, poverty, lack of family visits. Identifying the contexts with villains. Explain that villains come from somewhere (inside and outside the correctional center), e.g., from home or past trauma. Have youth identify where their villains come from.
3	Combating the villains: Exploration of strategies	How to combat the villains (risk factors) in our lives	Strategies to defeat the villains in our lives. Remind that resilience requires co-operation with the context. Identify available and accessible resources such as culture and religion (prayer), or attachment (such as a friend/teacher/correctional officer—relational resources)
4A/4B	The superhero team: Exploration of protective factors	Identify protective factors and resources Identify resources and access resources with invited guest.	Identify protective and supportive resources such as connectedness to ecological resources like community leaders, chaplains, religion, a family member. Practically engage with available and accessible resources. A community leader, pastor, teacher, social worker, can all be invited to inform youth about how they are resources—a Resource Expo.
5	Superhero strengths: Exploration of internal resiliency factors	Exploration of internal resiliency factors	Name internal strengths. These can be emotional, cognitive, and/or spiritual, such as perseverance, reflection, a positive attitude, and prayer. In keeping with the superheroes theme, these internal resilience factors can be considered individual superpowers, just as each superhero has their strengths. Identify context where they were used. Heroes narrate when last they remember themselves using these superpowers
6A/6B	My Superhero Story: What I have learned	The Traffic Light to help create a resilience plan Explore how we can develop and perform a play based on the social ecology of resilience	Create a resilience plan using the Traffic Light. Red: what I will STOP doing; YELLOW light: what you will do less of; and GREEN light: behaviors that will continue going forward Explore what has been learned. Highlight that each participant has personalized experiences and ways in which they have dealt with adversity. Probe for new ways they have learned in this program Develop a resilience play. The objective of this play is to allow youth to thread together the lessons about resilience, such as the context, significant adversity, resources, and their individual roles, as well as how they think they can best communicate what they have learnt to others. If the youth have access to a drama teacher, the teacher can help in this exercise. Potential audience members include COs, community leaders, other youth and family members.
7	Superhero celebration	Performance of the resilience play	Perform the play Perform graduation and awards ceremony

The program outcomes included contributing to the understanding of resilience among at-risk youth, identifying the factors that hinder and those that contribute to the resilience of youth in different contexts, and lastly to identify and utilize available and accessible resources to support resilience. The program structure presented above was implemented with the help of two program guides: a guide for the program presenter and a guide for youth. Each participant received a copy of the guide for youth used for the duration of the program, consisting of seven sessions. The program structure is expanded upon in the guides regarding each session’s needed materials, lesson content and focus, icebreakers, group activities, and outcomes.

The program presenter was trained by the program developer, as will be required for any future presenter of this program. The presenter must also be particularly knowledgeable on resilience, specifically, the social-ecological theory of resilience (Ungar et al., 2014). Skills such as presentation skills, listening skills, communication skills, and organizational/coordinating skills are recommended.

The program further encourages continued awareness of the difference between superheroes and real life, good and bad role models, responsibility, and false responsibility. The final session involves youth performing a play/dramatization of resilience to an audience constituted by their various ecologies and an awards ceremony and receipt of a certificate of participation.

### Aim

This study aimed to evaluate the resilience intervention program described in this article by exploring and describing the experiences of at-risk youth in a correctional center in Eswatini who participated in the program.

## Materials and methods

### Research design

This study utilized a qualitative, explorative-descriptive research design to provide rich, thick, and context-informed data (Creswell and Miller, 2000). The program was presented to 16 youths at a correctional center in Eswatini, referred to as End Gate (a pseudonym for ethical purposes) and who participated in phase 3 as described above.

### Participants and sampling

The recruitment of participants followed institutional ethical approval from the [excluded for review] and permission from His Majesty’s Correctional Services. Program participants between and including the ages of 15 and 25 volunteered participation. Participants were purposively sampled (Etikan et al., 2016), and those below the age of 18 provided individual assent following guardian consent for their participation. Participants older than 18 years signed informed consent forms. Additionally, participants had to be sufficiently fluent in and read and write English or SiSwati since these are the only two official languages of Eswatini and the research documentation was in these languages. The names of youth who participated in phase 3 (described above) were put in a container and drawn to make up the participants for program—the ideal size (16) was informed by the development of the program such as individual and group activities therein, as well as COVID-19 restrictions at the time. The youth who were not selected will be allowed to participate in the program when presented at End Gate after the research has been concluded.

The demographic profile of participants was all single Black Africans of which the majority were male, between the ages of 17–25 years and in grades 10–12.

The program presenter, a clinical psychologist, was involved in phases 2 and 3 and was also engaged in three training sessions with the first author, who familiarized and educated them on aspects of the program as outlined in Table 2. The presenter was not involved in the data collections of the participants’ experiences concerning the program.

### Data collection

To gather data on participants’ experiences, a World Café session was conducted (Brown and Isaacs, 2001). The World Café’s ability to open spaces for group dialogue, sharing, and linking ideas contributing to knowledge creation, made it ideal to gather feedback (Brown and Isaacs, 2001). Furthermore, the World Café accommodates different ages and cultures, which earns its suitability across contexts (Wheatley, 2005). The World Café process follows seven principles (Brown and Isaacs, 2001), namely: Setting the context; creating a hospitable space; exploring questions that matter; encouraging everyone’s contribution; cross-pollinating and connecting diverse perspectives; listening together for patterns, insights and more profound questions to build on unique ideas; and sharing collective discoveries—which allowed the authors to summarize what had been collectively shared regarding each question, as was indicated in the informed consent and—assent forms. The World Café data were analyzed, and the findings were refined and adapted to constitute the final program. The World Café method was suitable for the research site, End Gate, which had ideal spaces and allowed entry of all required World Café materials. The interview schedule used during the World Café session was reviewed by a panel of qualitative experts before use and was as follows: How did you experience the preliminary resilience intervention program? What was helpful for you in the preliminary program? What was your expectation from the preliminary program, and did the program satisfy those expectations? How do you recommend we improve the preliminary program when presented to future youths in correctional centers?

The presence of COVID-19 necessitated the adherence to the safety guidelines of the World Health Organization and, specifically, Eswatini’s health guidelines with regards to the pandemic. This adherence was also in line with the non-maleficence ethical obligation toward participants, and therefore COVID-19-related amendments were submitted for institutional approval. Additionally, HMCS was already practicing COVID-19 safety protocols such as wearing masks, hand washing/sanitizing, bi-weekly sanitizing of buildings, and social distancing. These safety measures were maintained throughout the research.

The first author took field notes during the World Café to capture responses regarding the World Café outputs during collective discovery. These notes provided clarification and an accurate understanding regarding inputs from participants and were included as data.

### Trustworthiness

Trustworthiness, referring to study robustness (Elo et al., 2014), was achieved through credibility, dependability, confirmability and transferability (Lincoln and Guba, 1985). Credibility was ensured through the use of reflexivity (being honest, critical, and reflective during the process and keeping reflective field notes); member checking (the World Café session allowed the first author and participants to ensure they had shared an accurate understanding of data); peer examination/review (experts critically reviewed the research); and structural coherence (integration of literature to highlight any difference or similarities from other researchers as well as providing linkages with other findings). Dependability was achieved through the dense description (detailed descriptions of the entire research process), code-recorder procedure (the independent coding of data with a co-coder and a consensus discussion afterward to discuss coding themes) and audit trail (keeping logs and field notes). Confirmability was ensured through keeping an audit trail and researcher reflexivity. The researcher ensured transferability through the dense description and the use of a purposive sample that allows other researchers to deliberately choose a similar and willing population to assess the applicability of findings (Etikan et al., 2016).

### Ethical considerations

The program was presented as a preliminary program on an actual sample of youth participants in a correctional facility. Participants were informed in the first session that they can withdraw from the program at any time and that there would be no adverse consequences. Parts of the program deal with sensitive information and, as such, there is potential for emotional distress. Therefore, it is essential to identify a qualified and accessible professional—before program implementation—if any adverse reactions result from participation in the program. A qualified psychologist was identified within the correctional center in the event distress was experienced from participation. During this study there were no incidences.

### Data analysis

The collected data were analyzed using thematic analysis and the six phases described by Braun and Clarke (2006), namely: familiarization with data, generating initial codes, searching for themes, reviewing themes, organizing themes, and producing a report. The first author and an experienced co-coder independently carried out initial thematic coding, and then the independently identified themes were discussed to reach consensus.

## Results

Table 3 provides a summary of the themes and sub-themes. The World Café is a collaborative conversational exercise of which outputs integrate participant contributions to inform themes and verbatim quotes are therefore not presented per individual contributions in the results.

**TABLE 3:** Overview of findings.

Themes	Sub-themes
Theme 1: Positive outcomes of participation	Recognition of the need for behavior change Importance of utilizing resources Lessons on positive reflection and acceptance Mental and emotional relief
Theme 2: Challenges encountered during the program	
Theme 3: Recommendations for the program	Involve more ecologies Increase program duration and frequency

The findings consist of three themes: positive outcomes of participation, challenges encountered during the program, and recommendations for the program.

### Theme 1: Positive outcomes of participation

The program had direct benefits and beneficial aspects for those who participated. The four sub-themes that constitute this theme are explored below.

#### Sub-theme 1: Recognition of the need for behavior change

Participants highlighted that the program encouraged them to appreciate the need for behavior change regarding problem-solving and decision-making through patience, cultivating positive habits, focus and perseverance—including post-incarceration:

*“Patience is a way to handle every situation because you can think before.”*

*“Knowing what you want in life, not being everywhere.”*

“…*when you fall down you need to figure out a way to bounce back.”*

These behavioral changes were also applicable to the context outside correctional facilities:

*“Expected to be taught on how to behave when we get out of here, so now I know some ways.”*

This is significant in the context and role of correctional centers that have the objective of rehabilitated individuals at time of release.

#### Sub-theme 2: Importance of utilizing resources

Participants demonstrated an understanding of the importance of, and their role in, identifying and utilizing available and accessible resources—including seeing themselves as resources:

*“Knowing where to get support.”*

“…*things are possible in life if you put in effort in what you do and ask for help if it is hard.”*

Furthermore, youth identified themselves as potential resources for others,

*“I learnt a lot that I want to teach my friends.”*

and they named immediately available and accessible resources, as well as other desired resources:

*“We must talk more with the sirs and madams, so they know what we need.”*

“…*we need [correctional authorities] to bring people like you here to teach us about life*…”

#### Sub-theme 3: Lessons on positive reflection and acceptance

Participants reported that the program contributed to positive realizations such as self-acceptance, awareness of risks, identifying internal protective factors, and a positive outlook:

*“It helps us to accept the things we can never change in life.”*

Additionally, youth took ownership of their history and demonstrated knowledge of the inevitability of risk factors:

“…*we should not let the past determine our future.”*

*“We learnt that risks can block the journey to victory, but we should be resilient, and face them.”*

Lastly, youth experienced a positive perception of themselves:

*“We are stronger than we think.”*

*“It boosted my self-esteem.”*

#### Sub-theme 4: Mental and emotional relief

Youth highlighted that the program provided cathartic release regarding their psychological distress, mental health conditions, and general sense of connection:

*“Therapy session I got it here because it is what I needed most to do well in life.”*

*“The programme was a therapy session because some who are here are here because of depression.”*

Participants further experienced cognitive relief, a sense of connection and hope:

*“It helps us to relax our minds and not think too much.”*

*“I felt like I finally found people I can share my feelings with, someone who understands us.”*

“…*we were treated like young people and given hope.”*

Psychotherapy is not part of the program, but the program is mindful of the potential need due to the sensitive material covered in some sessions. Therefore, the program can function as a bridge toward acknowledging the need for help and reaching out for therapeutic help if necessary.

### Theme 2: Challenges encountered during the program

This theme focused on what was experienced as obstacles during the preliminary program.

One of the obstacles experienced, specifically by female participants, were time constraints due to transport issues:

*“Maybe we can have a girl’s only programme because transport then limits our time.”*

This challenge refers to the fact that female participants reside at a different correctional center from the research site, End Gate, and a strict transport schedule was maintained. Owing to security reasons, the presence of a correctional officer, though positioned outside the actual room, during the programme contributed negatively in that youth felt censored in the level of expression:

*“It’s like [correctional officers] will hear and you don’t know what will happen.”*

*“Sometimes [correctional officers] are a risk, like now some things I can’t say.”*

The correctional officer signed a confidentiality agreement before the presentation of the preliminary program and this was explained to participants.

### Theme 3: Recommendations for the program

This theme focused on what participants felt could be added, changed, or improved in the preliminary intervention program.

#### Sub-theme 1: Involve more ecologies

The youth felt that the program needed to involve more participants generally from various contexts such as families, schools, communities and businesses:

*“The situation we are facing, we are not the first or the last, so you must teach more people.”*

“*To add more people in the class to find more different ideas.”*

and these were identified from different ecologies such as businesses, schools, the family, and the community:

*“To encourage more people in our community or in school to join (the programme) so they know.”*

*“My family does not know resilience, so they must be involved in the programme.”*

*“To do competition with other schools after the programme.”*

In the context of Eswatini, competitions between schools, in the form of sports, debates, science, function as resources for potential recruitment and transfer to better-resourced schools and access to potential sponsors and/or employers.

#### Sub-theme 2: Increase program duration and frequency

The youth highlighted the need to increase the entire program duration, their desire to function as resources (sharing knowledge gained), and the inclusion of more ecologies in the program:

*“The programme needs more time together*…”

The increase in the frequency of the programme was also encouraged:

*“To continue with the programme every year.”*

Youth proposed programme implementation in contexts outside the correctional setting such as schools and communities:

“…*so [the programme] must happen a lot with different groups.”*

Lastly, at the individual level, youth suggested that they can function as mentors to other youth:

*“Some of us must attend more groups, so we become peer educators when you guys are gone.”*

However, this is ideal for future program participants and *only* under the supervision of the program presenter.

## Discussion

This study aimed to evaluate a preliminary resilience intervention program by exploring and describing participants’ experiences, namely at-risk youth in a correctional center in Eswatini who participated in the program. Generally, similar to previous research (Soydan, 2010; Calitz, 2018), the participants experienced the program positively and found it beneficial. The program helped youth to acknowledge the need for them to engage in positive behavior change. Additionally, the positive experience of the program included youth positively reflecting on themselves, their past and acceptance of challenges and the need to deal with those challenges. Vasquez (2000) reported similar findings with a correctional population who participated in a resilience program that focused on ways to succeed in the face of adversity, counter self-sabotage, and generally lead productive lives. Post-program participation, participants seemed to accept accountability for their behaviors and could identify their strengths. The preliminary program appears to have influenced youth, through group interaction, identifying internal and external risk factors to resilience, shared experiences, and homework to engage in self-introspection, which, by extension, allowed them to identify risks inherent in their past and current lives—which may have influenced the positive change.

The program is grounded in the social-ecological theory of resilience, highlighting the positive significance of environments that avail and make accessible resources (Ungar et al., 2014). The participants realized the importance of accessing and utilizing resources such as correctional, family and community members. This echoed similar findings where youth could create and use interpersonal relationships resourcefully after a resilience intervention (Davis and Paster, 2000; Chandler et al., 2015). One possible explanation for this is that the program has a session that encourages team cooperation, resource identification and practical involvement of those who function as resources in their context. Correctional and community members participated in a “resilience expo” session and educated youth about their different roles and how and where they could be accessed. It is possible that the practical engagement sensitized youth to available resources and the benefits of utilizing these.

Another possible explanation for this is the Swati sociocultural practice that young people should not directly access and address adults—they need a person to function as a go-between to give depth and sincerity to whatever resource they require (known as *kuchutjwa*). As a result, youth may decide not to initiate communication regarding resource access due to the logistics involved. The significance of the role of culture in resilience has been highlighted by Theron et al. (2014), particularly how accounting for culture is protective to resilience. Additionally, the last session, which was a dramatization of resilience with corrections, family and community members as the audience, possibly anchored the significance of support from others in carrying out tasks or facing challenges. Mampane (2017) also found the involvement of family and school contexts in a resilience intervention program to have strengthened resilience, while others have found family connectedness to be protective to resilience (Poon et al., 2012).

The correctional population is known to be vulnerable to adversity (Bella et al., 2010) and mental disorders (Bochenek, 2016)—which has also been reported within the juvenile offender population of Eswatini (Malindisa and Winterdyk, 2015). Psychological and emotional relief was another direct positive outcome for participants. The program has sessions with individual and group activities that encourage identifying internal risk/protective factors that may have helped youth name and verbalize past traumas and the emotions attached. Furthermore, learning of shared psychological distress and adverse experiences possibly contributed to feeling relieved as participants realized they are not isolated or thinking that no one will understand them. Other resilience intervention programs have been found to buffer outcomes of traumatic experiences (Davis and Paster, 2000) and highlighted the need for mitigation of traumatic experiences as these are correlated to recidivism (Langa, 2007). It appears the program provided youth with a space for catharsis—through therapy is not part of the program—most likely due to the nature of the material covered and discussions about all ecologies that interact with youth. In addition, the empathic and respectful way the facilitator engaged with youth seems to be another factor that created the experience of a safe space for youth to share their adverse experiences and feel valued—as contributors to and creators of knowledge (Brown and Isaacs, 2001).

The challenges experienced during the program included time constraints, particularly for female participants due to a strict transport schedule and the presence of a correctional officer for security reasons. The transport schedule appears to have presented a challenge in that female participants would have to leave the day’s session maybe during a discussion that would emanate post the scheduled session activities—which, they felt, they wanted to be a party to, even though they were assured the day’s intended activities were completed. Female participants further proposed a girls-only resilience intervention program for this reason. The correctional setting requires a correctional officer to ensure security in any engagements with their population. This security measure presented a challenge in that, at times, the youth felt they had to be careful in what they shared for fear of consequences; whether these are real or imagined is uncertain in the event they were overheard. To counter this challenge, the correctional officer signed a confidentiality agreement, and this was explained to participants. Additionally, the correctional officer was positioned outside the room’s door where the program was presented.

Youth recommended that the program participants should also be from other ecologies external to the correctional setting. This recommendation is in line with authors who have consistently indicated that resilience buffering or protection cannot be divorced from contexts (Skovdal and Daniel, 2012; Markson et al., 2015) and, mainly, resource access and availability (Ungar et al., 2014) that is culturally meaningful (Theron et al., 2014). It appears that the preliminary program allowed youth to identify and value the role of their ecologies and that their resources were not limited to the correctional setting. This finding echoes studies that indicated that increased caregiver/parent involvement is protective to resilience, as well as the experience of meaningful interpersonal relationships (Eisenberg and Resnick, 2006; Steyn and Louw, 2012).

Lastly, participants recommended an increase in the duration for each session and to regularly provide the program across their population. This is possibly an indication that youth found the program to be helpful. Additionally, this could also be linked to the significance of interpersonal relations, such as family support, as resources protective to their resilience which the program informed them about. Furthermore, a session helped youth identify their positive coping mechanisms—an approach advocated by positive psychology (Cohn and Fredrickson, 2010). This contributes to a positive self-image which is a positive outcome, particularly in the under-resourced correctional context, which compromises rehabilitation and development (Barnert et al., 2016; Atilola et al., 2018).

## Conclusion

The findings indicate the potential value of the program for youth in corrections in Eswatini in that they shed light on youth’s experiences thereof, which underscores the need for, and the importance of resilience-focused intervention programs tailored for the juvenile correctional population. The findings will also be applied to improve the program prior to further presentation. Therefore, the program can be applied in correctional facilities in Eswatini to intentionally buffer, promote, and protect incarcerated youth’s resilience, which can contribute positively to rehabilitation.

A limitation of the study is that the participants were of a homogeneous race and excluded other age groups, particularly those below 15 years, within the juvenile correctional population. It is recommended that future studies involve other age groups within the juvenile population. Furthermore, there was only qualitative data collection regarding evaluation of the program. Future studies can focus on quantitative evaluation and the longitudinal effect. Research focused on similar programs for parents/caregivers, families, communities, and other ecologies such as schools, which meaningfully interact with youth, help with resource-creation, and access and availability, is recommended.

## Data availability statement

The raw data supporting the conclusions of this article will be made available by the authors, without undue reservation.

## Ethics statement

The studies involving human participants were reviewed and approved by Health Research Ethics Committee of the North-West University. Written informed consent to participate in this study was provided by the participants’ legal guardian/next of kin.

## Author contributions

All authors listed have made a substantial, direct, and intellectual contribution to the work, and approved it for publication.
